# New Therapeutics for HCC: Does Tumor Immune Microenvironment Matter?

**DOI:** 10.3390/ijms24010437

**Published:** 2022-12-27

**Authors:** Arshi Khanam, Shyam Kottilil

**Affiliations:** Division of Clinical Care and Research, Institute of Human Virology, University of Maryland School of Medicine, Baltimore, MD 21201, USA

**Keywords:** hepatocellular carcinoma, tumor immune microenvironment, immunotherapy

## Abstract

The incidence of liver cancer is continuously rising where hepatocellular carcinoma (HCC) remains the most common form of liver cancer accounting for approximately 80–90% of the cases. HCC is strongly prejudiced by the tumor microenvironment and being an inflammation-associated condition, the contribution of various immune mechanisms is critical in its development, progression, and metastasis. The tumor immune microenvironment is initially inflammatory which is subsequently replenished by the immunosuppressive cells contributing to tumor immune escape. Regardless of substantial advancement in systemic therapy, HCC has poor prognosis and outcomes attributed to the drug resistance, recurrence, and its metastatic behavior. Therefore, currently, new immunotherapeutic strategies are extensively targeted in preclinical and clinical settings in order to elicit robust HCC-specific immune responses and appear to be quite effective, extending current treatment alternatives. Understanding the complex interplay between the tumor and the immune cells and its microenvironment will provide new insights into designing novel immunotherapeutics to overcome existing treatment hurdles. In this review, we have provided a recent update on immunological mechanisms associated with HCC and discussed potential advancement in immunotherapies for HCC treatment.

## 1. Introduction

Hepatocellular carcinoma (HCC) remains one of the most common manifestations of primary liver cancer, accounting for approximately 80–90% of the cases [1]. Diverse factors including gender, race/ethnicity, chronic viral hepatitis, heavy alcohol use, cirrhosis, inherited metabolic diseases, diabetes, obesity, and chronic toxin exposure increase the risk of HCC [2,3,4,5]. However, it most often occurs in people suffering from chronic liver diseases (CLD) primarily caused by viruses, such as hepatitis B and hepatitis C, and alcohol, though the ultimate outcomes are nearly similar in all HCC patients. Since the mechanism of cellular and molecular pathogenesis differs depending on the diverse etiologies and genotoxic insults, further understanding of the pathophysiology is warranted for the advancement of new therapeutic interventions into clinical practice. Recently, both HBV- and HCV-associated risk of HCC development has decreased tremendously due to the HBV vaccination and effective anti-viral therapies [6]. Change in dietary habits and lifestyle, for instance, abstinence from alcohol in chronic alcohol drinkers, may decrease the risk of HCC [7].

The liver is a unique organ with the ability to regenerate itself after acute damage as the differentiated hepatocytes re-enter in the cell cycle substituting the damaged hepatocytes [8]. In chronic liver damage, continuous activation of the immune cells in response to different stimuli triggers liver inflammation affecting hepatic architecture including hepatocytes and biliary epithelial cells, and simultaneously induces reactive oxygen species (ROS) production and deoxyribonucleic acid (DNA) damage, augmenting the occurrence of DNA mutations and increasing the fibrosis that associates with HCC carcinogenesis [9,10,11]. In general, liver inflammation is counterbalanced by the host regulatory mechanisms; however, in the event of failed regulatory mechanisms, sustained and non-resolving inflammation occurs, which plays a pivotal role in the development and progression of HCC. HCC represents a typical prototype of inflammation-associated cancer, as most of the cases emerge in the setting of hepatic injury and inflammation [12]. The HCC-linked inflammation can be instigated and propagated by the extraneous pathways through the activation of different pattern recognition receptors (PRRs) by the pathogen-associated molecular patterns (PAMPs) derived from gut microbiota or damage-associated molecular patterns (DAMPs) released from the dying cells, whereas the intrinsic pathway including genetic events stimulate the inflammation-associated programs directing the establishment of an inflammatory microenvironment [12]. Both pathways turn on the different transcription factors including nuclear factor kappa B (NF-κB) and signal transducer and activator of transcription (STAT3) that serve as main drivers of the inflammatory response by enhancing the production of inflammatory cytokines and chemokines. Ground-breaking technologies including single-cell RNA sequencing established the presence of an extremely heterogeneous immune cell population in liver cirrhosis and HCC [13]. Hepatic tumor immune microenvironment constitutes a complex mixture of immune cells, such as neutrophils, Kupffer cells, myeloid derived suppressor cells (MDSCs), regulatory dendritic cells (Dregs), and regulatory T cells (Tregs) including other cell types. Their incongruous activation contributes to the regulation of tumor growth, metastatic potential, and outcome after therapy. Moreover, the tumor itself participates in the inflammatory process by secreting different factors, leading to the recruitment of inflammatory cells that assist in the build-up of a tumor-supportive microenvironment [14]. Cellular studies reported the significance of the tumor microenvironment (TME) in predicting the outcome in HCC patients [14]; therefore, extensive characterization of the hepatic immune microenvironment comprising complex cellular networks will incite the development of novel therapeutic interventions against HCC.

For the successful treatment of primary liver cancer, it is critical to comprehend inflammation-triggered events. Indeed, the past decade was revolutionary in terms of cancer treatment, majorly focusing on the targeting of precise molecules using antibody-based immunotherapies that modify immune responses against tumors, challenging the use of conventional (chemotherapy and radiation therapy) therapies [15]. Although immunotherapies have shown substantial clinical benefits in those who establish durable responses, they still impose significant challenges in patients who have lower response or do not respond to therapy at all. Adoptive immunotherapy in conjunction with multi-kinase inhibitors are being widely investigated due to their curative potential and extended survival in HCC patients [16,17]. Here, in this review, we have provided an update on hepatic tumor immune microenvironment (TIME) and elaborated on how it is associated with HCC pathogenesis and can be further targeted for therapeutic interventions.

## 2. Cellular Component of the Hepatic Tumor Microenvironment

Organ microenvironments are irrefutable modules enabling the proliferation of cancer cells to organ systems exterior to the primary tumor. The hepatic tumor immune microenvironment constitutes cellular and non-cellular components including a complex mixture of immune cells [18]. These cellular components yield the non-cellular components of the tumor stroma comprising different growth factors, proteolytic enzymes, and their inhibitors along with inflammatory cytokines. The profound understanding of tumor immunology research discovered that the tumor microenvironment (TME) is constantly modulated by the genetic alterations, epigenetic factors, cellular metabolism, and impaired oncogenic signaling, imposing significant challenges in developing standard treatment plans.

### 2.1. Tumor-Associated Neutrophils: New Felon in HCC Tumor Immune Microenvironment

Among other myeloid cell populations that are generated in the bone marrow, neutrophils are the most abundant leukocytes present in the circulation and are the first to be recruited at the site of infection or injury, where they act as a first line of defense. Emerging data educate that neutrophils are critical for tumor initiation, proliferation, vascularization, migration, and invasion through several mechanisms, ranging from direct support to tumor cell survival, causing immunosuppression and genetic mutations [19]. Infiltrating tumor-associated neutrophils (TANs) display anti-tumorigenic or pro-tumorigenic phenotypes. At the early stage of HCC, neutrophils are primarily located at the periphery of the tumors and exhibit anti-tumoral phenotype, whereas at the later stage, their pro-tumoral properties dominate, supporting cancer progression [20]. The pro-tumoral or anti-tumoral phenotype of neutrophils in the primary tumor or metastatic site is highly reliant on the cytokine milieu. The anti-tumorigenic neutrophils are classified as N1, while the pro-tumorigenic neutrophils are categorized as N2 phenotypes [21]. N1 neutrophils direct tumor cell cytotoxicity, constraining the development of cancer, while N2 TANs endorse cancer development through supporting carcinogenesis, angiogenesis, tumor growth, and metastasis by destroying anti-cancer immunity and synthesizing neutrophil extracellular traps (NETs) [22,23]. Establishment of NETs is initiated by the innate immune receptors through downstream intracellular mediators comprising ROS, which are generated by NADPH oxidase or mitochondria, activating myeloperoxidase, neutrophil elastase, and protein-arginine deiminase type 4 (PAD4) to promote chromatin decondensation [24,25]. NETs release occurs primarily via a cell death process called NETosis; however, live cells can also trigger the release of NETs. Systemic effects of tumors modulate NETosis, instigating NET-associated complications in cancer. Upregulation of granulocyte colony stimulating factor (G-CSF) in HCC increases systemic NETosis and transforms HCC into a high-grade phenotype [26,27].

The presence of G-CSF and tumor necrosis factor-alpha (TNF-α) in the pro-tumoral area induces the immunosuppressive profile in neutrophils by augmenting programmed death-ligand 1 (PD-L1) expression [28]. Moreover, cancer-associated fibroblasts (CAFs) infiltrating HCC stimulate the activation and survival of TANs, shown by higher expression of CD66b, PD-L1, CXCL8, TNF-α, and chemokine ligand 2 (CCL2), where enrichment of CD66b+ neutrophils in the peritumoral region is associated with decreased overall survival of patients [29]. CAFs secrete cardiotrophin-like cytokine factor 1 (CLCF1) inducing the production of CXCL6 and transforming growth factor-β (TGF-β) by tumor cells deriving TANs polarization into N2 phenotype and promoting tumor stemness [30]. CLCF1-CXCL6/TGF-β axis supports the recruitment of N2 TANs along with the regulation of cancer stemness, contributing to the poor prognosis of HCC patients. HCC-CAF-primed neutrophils suppress T cell activity by efficiently suppressing the T cell proliferation and IFN-γ production mainly through neutralization of PD-L1, which attenuated T cell suppression mediated by CAF-primed neutrophils, confirming that neutrophil mediates immune suppression through programmed death PD-1/PD-L1 pathway. In the HCC microenvironment, neutrophils act as a primary source of MMP-9 encouraging angiogenesis by the release of pro-angiogenic factors [31]. Furthermore, TANs secrete CCL2 and CCL17, which correlate with tumor size, microvascular invasion, extent of tumor differentiation, staging, and poor survival time [32].

Neutrophils in intratumoral regions express autophagy-specific protein LC3 and autophagosomes, deriving the continued production of pro-metastatic oncostatin M and MMP9, which advances the migration of cancer cells [33]. TME delays apoptosis in neutrophils with retained Mcl-1 and low levels of cleaved caspase-3. Inhibition of autophagy by an autophagy-specific inhibitor 3-methyladenine (3-MA) suppressed the production of these molecules and eliminated TME-mediated neutrophil survival, indicating a pro-tumorigenic role of neutrophil autophagy. Importantly, depletion of neutrophils effectively inhibited tumor angiogenesis and growth, suggesting a critical role of neutrophils during HCC [33].

### 2.2. Regulatory Dendritic Cells: Another Feather in the Cap of Immune Suppression during HCC

Dendritic cells (DCs) are the professional antigen presenting cells, capturing the pathogen or tumor-derived antigens, presenting them to the naïve T cells to generate effective immune response by differentiating them into effector T cells; however, these functions are generally weakened in the tumor microenvironment [34]. Due to the overwhelming growth, cancer cells alter their metabolism to uptake higher amounts of nutrients in tumor biomass and generate new tumor cells [35]. This aberrant tumor cell metabolism, in turn, induces extensive modifications in TME including tryptophan depletion, lactate and lipid accumulation, hyper glycolysis, and acidification, which minimizes the function of DCs leading to the incidence of tumor immune escape [36]. In fact, DCs present in TME block anti-tumor immunity by encouraging cancerous cell growth and dissemination, inducing genomic damage and advancing neovascularization. In most tumors, these cells preferentially acquire regulatory phenotypes known as regulatory dendritic cells (Dregs), that primarily serve tumor cells to escape immune mechanisms by expressing diverse immune regulatory molecules and producing higher levels of immunoregulatory cytokines, and have minimal aptitude to encourage T cell activation and proliferation [37]. Existing literature reveals that tumor-derived factors and tumor itself can transform conventional or myeloid dendritic cells into Dregs [38]; however, whether non-tumor cells in TME can also encourage Dregs differentiation is not clear. Dregs directly or indirectly maintain T cell unresponsiveness by limiting T cell polarization, Tregs, and MDSCs differentiation through the induction of indoleamine 2, 3 dioxygenase (IDO), IL-10, TGF-β and express COX-2, iNOS and arginase, affecting specific microenvironmental conditions in premalignant niches [37,39,40]. Production of IDO is triggered by vitamin D and prostaglandin E2 (PGE2), while its transcription is regulated by the signal transducer and activator of transcription 3 (STAT3), initiated by IL-6 from the tumor cells [41]. It has been proven that IL-6-treated DCs downregulate CD1a, CD83, CD86, HLA-DR and upregulate CD14 expression, inducing Dregs [41]. IDO degrades the essential amino acid tryptophan, required for protein synthesis, leading to the build-up of its metabolite, the kynurenine [42]. Whereas depletion of tryptophan may lead to the inhibition of T cell proliferation and accumulation of its metabolite may employ immune cell toxicity and enhance IDO production in DCs, leading to DC-induced immunosuppression and further contributing to tumor evasion [43]. The existence of CD14+ DCs was observed in peripheral blood and tumor tissues of HCC patients irrespective of tumor stages. These cells express inhibitory molecules including PD-1 and cytotoxic-lymphocyte antigen 4 (CTLA-4) on their surface and produce IL-10 in response to lipopolysaccharides (LPS) neutralization, which weakens the immune-suppressive effect of CD14+ DCs, suggesting that the immunosuppressive effect of these cells is driven by inhibitory molecules and IL-10 [44].

Additionally, tumors redirect the process of dendropoiesis (DCs generation) and further polarize these cells into a phenotype that commonly blocks the development of anti-tumor immunity [45], further backing tumor progression by allowing intra-tumoral neoangiogenesis and metastases. DCs capacity to direct the immune response is not an intrinsic feature; rather, it arises due to specific microenvironmental signals originated from TME, such as local cytokine milieu and other soluble factors. For instance, tumor-derived IL-10, TGF-β, and PGE2 can manifest DCs to attain regulatory rather than stimulatory functions, where IL-10 appeared most tolerogenic compared to other factors since it inhibited the maturation of these cells, restricted the expression of MHC class I and II and costimulatory molecules, and alleviated the production of inflammatory cytokines [46]. Studies reported that bone marrow-derived DCs cultured in the presence of IL-10 presented low levels of MHC class II, CD40, CD80, CD86, and IL-12p70 and abundantly produced IL-10, and were competent in expanding functional Tregs [47,48]. Peripheral blood and CD11c+ tumor-infiltrating myeloid cells express PD-1 in HCC in both humans and mice, which repressed IL-2 and IFN-γ as well as antigen-specific CD8 T cell proliferation under in vitro and in vivo settings, respectively; suggesting that immune surveillance against tumors is strongly regulated by PD-1 expression on DCs [49]. Given that Dregs are critical in deriving immunosuppression in HCC, new therapies selectively targeting immunosuppressive functions in DCs may assist in restoring immunostimulatory functions of DCs in TME.

### 2.3. Tumor-Associated Macrophages: One of the Gritty Culprit

Macrophages belong to the mononuclear lineage, originating and differentiating from myeloid progenitors and circulating monocytes. During the embryonic hematopoiesis, monocytes are recruited from the bone marrow reaching to different tissues and organs via traveling through circulation and then differentiating into tissue-resident macrophages, with exceptional plasticity and functional diversity. Nevertheless, one needs to bear in mind that not all the macrophages are derived from circulating monocytes including Kupffer cells (KCs) in the liver [50]. In recent years, one of the most important paradigm shifts in the macrophage biology is the origin of tissue macrophages. Earlier, it was believed that tissue macrophages are derived from circulating blood monocytes, while at present, it is clear that tissue macrophages are developed during the embryonic development, preserved separately from blood monocytes during homeostasis, and primarily self-renew from resident stem cells originated from fetal yolk sac [51]. Tissue-resident macrophages are a heterogeneous population that is inevitably required for performing tissue-specific functions and maintaining homeostasis. Fundamentally, macrophages are phagocytic in nature, retain antigen presentation capability, and provide non-specific defense by clearing foreign and harmful pathogens, cellular debris, and tumor cells, and further initiating specific-defense mechanisms [52]. Depending on the surrounding environment and cytokine milieu, macrophages can differentiate into classically activated macrophages (M1) and alternatively activated macrophages (M2), which can convert into each other owing to the changes in the internal environment. M1 macrophages are induced by T helper type 1 (Th1) cell signature cytokine IFN-γ and/or toll-like receptor (TLR) ligands, whereas M2 macrophages are encouraged through the stimulation of Th2-associated cytokines, such as IL-4 and IL-13 along with macrophage colony stimulating factor (M-CSF), TGF-β, and glucocorticoids [53]. Hepatic macrophages, referred to as KCs, can remove microbial products and other deleterious substances transported to the liver via the blood circulation. The hepatic parenchyma possesses approximately 80% of all macrophages of the body and is traversed by blood monocytes; however, this occurs only during liver injury. Normally, tissue-resident macrophages are acknowledged as M2-like phenotypes with a fundamental role in tissue homeostasis immune surveillance and resolution of inflammation by controlling excessive immune activation. Since the liver is persistently exposed to antigens from the gut and low levels of bacterial endotoxins, several mechanisms suppress incidental immune activation including M2 macrophages; therefore, the liver acts as an immunotolerant organ [54]. Tissue macrophages express pattern recognition receptors, such as toll-like receptors (TLRs), NOD-like receptors (NLRs), lectins and scavenger receptors that recognize a wide variety of PAMPs and DAMPs. Expression of heterogeneous receptors on macrophages indicates its involvement in the activation of different classes of immune response to different pathogens and viruses. Tumor-associated macrophages (TAMs) develop a pro-inflammatory microenvironment and are a potential source of steatosis-stimulated Wnt expression. The active Wnt/β-catenin signaling in macrophages may encourage the growth of tumor progenitor cells, increasing the risk of HCC as well as cholangiocarcinoma in obese individuals [55].

Tumor-associated macrophages secrete different cytokines, such as IL-6, IL-10 and TNF-α, chemokines CCL17, CCL22, CCL24, CXCL12, CXCL8 and growth factors TGF-β, vascular endothelial growth factor (VEGF), epidermal growth factor (EGF), fibroblast growth factor (FGF), along with other soluble factors, such as osteopontin, cyclooxygenase-2, and matrix metalloproteinase (MMPs), modulating tissue architecture and favoring tumor cell migration, invasion, cancer progression, and metastasis [56,57,58]. TME constitutes two types of TAMs containing tissue-resident and infiltrated macrophages. During HCC, most tumor cells support the infiltration of macrophages into the tumors by displaying higher glypican-3 expression where these cells play a bigger role in HCC progression by dictating various inflammatory lesions than those of resident macrophages, which produce chemokines, deriving leukocytes influx to induce inflammation [59]. Higher TAM infiltration is frequently correlated with poor clinical outcomes in various types of tumors and reduced response to standard cancer therapeutics including chemotherapy, radiotherapy, as well as targeted therapy; therefore, limiting their infiltration could be beneficial to overcome these obstacles [60]. However, TAMs are not deleterious completely; they confer protection by eliminating the tumor. Macrophages eradicate apoptotic cells and cell debris to support the recurrence of homeostasis during the resolution of inflammation and participate in the formation of vascularized granulation tissue, epithelization, and subsidizing scar formation to every stage of damage repair [61]. However, the moral functional abilities of TAMs in TME are dominated by their ruthless behavior.

### 2.4. Myeloid-Derived Suppressor Cells: Frenemy of HCC

In the recent decade, myeloid-derived suppressor cells (MDSCs) attracted increased attention in different cancers including HCC. MDSCs are pathologically activated cells that are involved in immune regulation in different disease conditions including chronic infections, auto-immunity, sepsis, and cancer, and are strongly associated with poor clinical outcomes, especially in cancer [62]. MDSCs are classified into two different phenotypes including granulocytic/polymorphonuclear MDSCs (PMN-MDSCs) and monocytic MDSCs (M-MDSCs) conforming to their origin from granulocytic or monocytic myeloid cell lineages [63]. Nevertheless, these cells share common biological features including upregulated expression of arginase-1 (arg-1) and STAT3, and induced endoplasmic reticulum (ER) stress. Whereas, they differ in other features, such as PMN-MDSCs mediate immune suppression favorably using reactive oxygen species (ROS), arg-1, and PGE2, while M-MDCs utilize nitric oxide, immunosuppressive cytokines including IL-10 and TGF-β and immunoregulatory molecules, such as PD-L1 [63]. The main characteristics delineating MDSCs is their strong immunosuppressive nature. These cells can control immune responses mediated by diverse immune cells including T and B cells, and NK cells. MDCSs are thoroughly dispersed in the bone marrow, spleen, and peripheral blood. The presence of MDSCs has been detected in the TME where they participate in the tumor growth and angiogenesis [64]. Therefore, MDSCs is a potential approach for enhancing the current treatment of cancers. During cancer, MDSCs acquire several changes in their phenotype, functions, and metabolic processes. It is well established that metabolic reprogramming occurs during cancer, which is essential for the tumor cells to withstand their high-energy requirement to accelerate their proliferation, differentiation, and survival. MDSCs struggle for the nutrients and oxygen in the TME and thereby acclimatize their metabolism in accordance with the TME [63]. MDSCs act vigorously, selecting the most competent metabolic pathways to endure their regulatory, suppressive, and pro-tumorigenic functions. Lipid, glucose, and amino acid metabolism are altered in MDSCs where lipid metabolism is crucial for its differentiation and functions, while glucose metabolism supports their maturation from bone marrow precursors [65,66]. In addition, upregulated glycolytic pathways defend MDSCs from apoptosis and participate in their survival by preventing ROS-mediated apoptosis. Under hypoxic conditions, the activation of hypoxia-inducible factor-1α (HIF-1α) triggers high glycolysis in MDSCs [67]. Overexpression of HIF-1α is highly associated in supporting tumor growth and metastasis by instigating angiogenesis and regulating cellular metabolism to overcome hypoxia. In the case of MDSCs, HIF-1α regulates the differentiation and function of MDSCs in the TME and, in fact, facilitates the differentiation of M-MDCs into tumor-associated macrophages [67].

The frequencies of circulating MDSCs are increased during HCC and directly correlate with the combative tumor features and volume [68]. Tumor-infiltrating leukocytes consisted of the highest percentage of MDSCs expressing PD-L1 than those present in the liver-infiltrating leukocytes and peripheral blood. Several lines of evidence pointed toward prognostic implications and translational significance of MDSCs in HCC. A study by Nan et al. identified a novel marker, LOX-1 (lectin-like oxidized low-density lipoprotein (LDL) receptor-1) in PMN-MDSCs in HCC patients and found that LOX-1+CD15+ cells were increased in the peripheral blood and their frequencies were associated with those present in HCC tissue [69]. These cells inhibited the proliferation of IFN-γ producing T cells under in vitro setting, while their LOX-1-CD15+ counterpart did not; confirming the role of LOX-1 in MDSC-derived immune suppression. MDSCs influence the tumor microenvironment by producing several angiogenic factors and vascular-modulating enzymes, such as bombina variegata peptide 8 (Bv8, a homolog of endocrine gland-derived vascular endothelial growth factor) through G-CSF dependent STAT3 signaling, promoting angiogenesis and hematopoietic cell mobilization. Additionally, MDSCs could obtain endothelial cell properties in TME, directly incorporating into tumor endothelium [70]. Reversing the pro-tumor effects of MDSCs could be attained by their depletion, inhibiting their trafficking and migration into TME, and restricting their immunosuppressive functions.

### 2.5. Regulatory T Cells: Persistent Suppressors of Anti-Tumor Immunity

Regulatory T cells (Tregs) are indispensable for maintaining peripheral tolerance by suppressing the immune response, which confines autoimmune diseases and chronic inflammatory diseases. However, they are also involved in the development and progression of tumors by restricting effective anti-tumor immunity, and are linked to poor survival in different cancers, serving as a critical target to explore [71]. The existence of two types of Tregs has been proven: (1) Natural Tregs (nTregs) and (2) Induced Tregs (iTregs) [72]. Naturally occurring Tregs develop as a distinct lineage in the thymus and specifically express forkhead box P3 (FOXP3) transcription factor in their nucleus and CD25 and CTLA-4 on their surface, maintaining immunological tolerance and homeostasis, while induced Tregs develop from naïve conventional T cells in the periphery after receiving an antigenic signal [72]. Few studies indicate that iTregs are CD4+ and FOXP3, whereas others suggest that both Tregs unanimously express FOXP3 and participate in immunological tolerance in circumstances where nTregs are reduced or functionally defective [73]. A significant amount of effort has been made to uncover the mechanisms of Tregs’ suppressive activity in tumor immunity. Generally, Tregs modify the effector cell function at different stages of the immune response. At an early stage, Tregs limit T cell activation and proliferation by the induction of genes involved in growth arrest or inhibition of cell proliferation, while at the later stage, they control the differentiation and function of effector cells by producing suppressive cytokines, inhibiting effector cell migration, and causing metabolic disruption [74].

**Figure 1:**
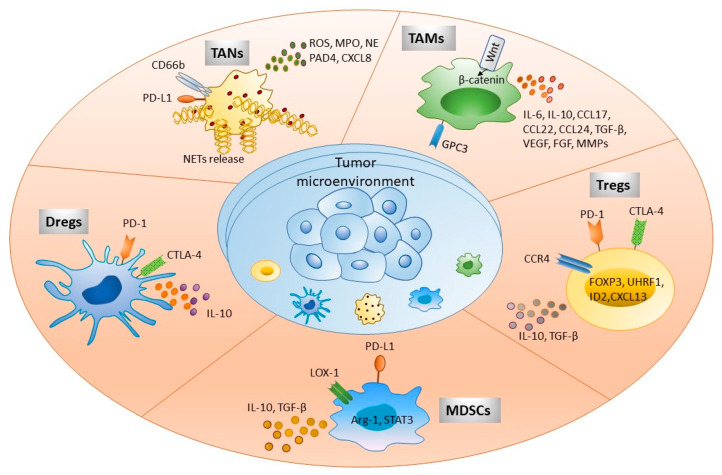
Immune cells present in tumor microenvironment participate in HCC development. Tumor immune microenvironment exhibits various immune cells as shown in the figure. These cellular populations interact with other parenchymal, nonparenchymal, and tumor cells directly by expressing different cell surface molecules or via secretory factors, such as cytokines, chemokines, and other soluble factors. This complex cellular interplay significantly participates in immune escape, tumor invasion, and metastasis. Targeting these molecules serves as a potential immunotherapeutic strategy to treat HCC patients. Abbreviations: FOXP3, Forkhead box protein 3; ROS, Reactive oxygen species; MPO, Myeloperoxidase; NE, Neutrophil elastase; PAD4, Protein-arginine deiminase type 4; NETs, Neutrophil extracellular traps; GPC3, Glypican 3; ILs, Interleukin; CCL and CXCL, Chemokine ligand; TGF-β, Transforming growth factor-β; VEGF, Vascular endothelial growth factor; FGF, Fibroblast growth factor; MMPs, Matrix metalloproteinases; CTLA-4, Cytotoxic T lymphocyte antigen 4; CCR4, Chemokine receptor 4; LOX-1, Lectin-like oxidized low-density lipoprotein (LDL) receptor-1.

Tregs target a wide range of immune cells, such as dendritic cells, macrophages, NK cells and neutrophils, CD4+, CD8+ T cells and B cells, and are suspected to have lysing effect on these cells. Chemokine ligands CCL17 and CCL22 present in TME facilitate the infiltration and accumulation of Tregs in tumor tissue through chemokine receptor 4 (CCR4), a G protein-coupled receptor, primarily expressed on the most immunosuppressive Tregs population. Recently, it has been reported that tumor-infiltrating Tregs (TIL-Tregs) are predominantly CCR4+ and are functionally more immunosuppressive than those of CCR4-Tregs [75]. Nearly 90% of TIL-Tregs are CCR4+, while peritumoral tissue and peripheral Tregs contain a significantly lower proportion of CCR4+ Tregs. Single cell gene expression database of tumor infiltrating Tregs (TIL-Tregs) exposed that these cells have higher expression of genes involved in Tregs proliferation and exhaustion, such as UHRF1, ID2, and CXCL13 [76], while CCR4-Tregs express granzymeH, LYN, CXCL9, and pro-melanin-concentrating hormone genes, which are involved in T cell differentiation and activation [77]. Adaptive immune resistance to immunotherapies is partly attributed to the upregulation of CCR4 ligands in the tumor, initiating increased Tregs migration to the TME. Checkpoint inhibitors, such as anti-PD-1 and anti-CTLA-4 antibodies, have provided exceptional anti-tumor responses and extended the survival of patients in several types of cancers; nevertheless, few of them fail to respond or if they do respond, they relapse later by developing resistance to immunotherapy, which is attributed to the accumulation of Tregs in the tumors [78,79]. Therefore, CCR4 antagonism is a potential approach to control Tregs accumulation in the TME. Indeed, CCR4 antagonism in combination with sorafenib showed enhanced anti-tumor efficacy by transforming the immune landscape toward anti-tumor immunity through overall reduction in Tregs, diminishing proportions of CCR4+ TIL-Tregs, decreasing PD-1+CD8+ T cells, and enhancing IFN-γ+CD8+ T cells [75]. Additionally, CCR4 antagonist reduced the tumor growth of CCL17^high^ and CCL22^high^ tumors, suggesting that CCR4 blockade is beneficial not only for controlling Tregs infiltration and reversing CD8 T cell exhaustion, but also for restricting tumor growth which can be particularly beneficial in those who develop sorafenib resistance. One of the main challenges of targeting tumor Tregs is to specifically reduce tumor Tregs without affecting the normal tissue and peripheral pools where they are still needed to perform their normal function and maintain immune tolerance. CCR4 inhibition with CCR4-351 is a promising target in that aspect as it appears to be very selective in averting Tregs migration into TME without affecting the other pools of Tregs present in peripheral blood, skin, and spleen. This finding most likely can be translated into humans where the tumor has higher CCL17/CCL22 levels since depletion of systemic Tregs has the risk of inducing autoimmunity [80]. Overall, the function of immune cells in HCC has been summarized in Figure 1.

## 3. Importance of Immune-Based Therapies in HCC

Immunological mechanisms are strongly associated with HCC pathogenesis; therefore, immune-based therapies are a critical target and indeed proven to be effective and safe in treating solid tumors, with tolerable toxicity and relatively long-term survival. Liver is an immunologically tolerant organ, preventing hypersensitivity to antigens and bacterial products; however, this tolerogenic property along with immunosuppressive TME culminates anti-tumor immunity against HCC [81]. Therefore, reversal of anti-tumor immunity is highly warranted. Cell-based therapies, checkpoint inhibitors, and combination immune therapies are widely investigated for the treatment of HCC.

### 3.1. Use of Cell-Based Therapies

#### 3.1.1. Adoptive Cell Therapy

In recent years, the introduction of numerous cell-based therapies to activate a patient’s immune system for restricting tumor growth has been witnessed. The use of adoptive cell therapy (ACT) which employs immune cells of patients or healthy donors to enhance anti-cancer immunity has gained substantial attention; although the initial attempts to develop ACT in HCC were unsuccessful in reaching clinical stage due to the absence of efficacy and comparative intricacy of the procedure [82]. In this technique, lymphocytes are sensitized to tumor antigens and/or expanded in the lab settings and then infused into the patients to enhance anti-tumor immunity [83]. Tumor infiltrating lymphocytes (TILs), peripheral blood T cells, lymphokine-activated killer cells (LAK), cytokine-induced killer (CIK) cells, and NK cells are used for ACT and have long-lasting anti-tumor effects. However, it is complex and considered highly individualized therapy as effector cells are primarily derived from the patients. At present, chimeric antigen receptor (CAR) T-cell therapy received approval from the FDA to treat different cancers [84]. Here, we have summarized different types of cell-based therapies, which can be used to treat HCC.

#### 3.1.2. CAR T-Cell Therapy

After years of painstaking research, CAR T-cell therapy emerged as a potential approach to treat hematological malignancies; however, their application in solid tumors is under development due to tumor heterogeneity, absence of specific targets, and vulnerability to tumor microenvironment. In this technique, T cells are genetically engineered to generate chimeric antigen receptors (CARs) on their surface, enabling them to identify and bind to specific tumor-associated antigens and proteins, which are present on the surface of the tumors for targeted killing [85]. Unlike conventional T cells, CAR T-cells do not require major histocompatibility complex (MHC) antigen presentation, circumventing tumor immune escape.

CAR T-cells have several targets, such as alpha-fetoprotein, glypican, and C-Met. Glypican-3 (GPC3) is highly expressed in HCC and used as an immunohistochemical biomarker [86]. It is a cell surface glycophosphatidylinositol-linked protein belonging to the heparan sulfate proteoglycan family with highly negative charges, playing a crucial role in cell growth, differentiation, and migration by functioning as a coreceptor to modulate Wnt/beta catenin signaling in order to promote cell proliferation in HCC [87]. GPC3 can be found in the circulation and tumor surroundings due to its release from the cell surface, influencing the efficiency of anti-cancer therapy, which specifically targets these antigens. Recombinant soluble GPC3 interferes with HCC cell growth, suggesting that GPC3 may compete with cell surface GPC3 to bind GPC3-interacting molecules or may block GPC3-targeted therapies. A recent study constructed two types of CAR T-cells targeting different epitopes of GPC3 and found that both human YP7 CAR T-cells and 32A9 CAR T-cells possess GPC3-specific anti-tumor functions and inhibit tumor growth under in vitro and in vivo settings [87]. However, sGPC3 markedly suppressed the release of cytokines and cytotoxicity of anti-GPC3 CAR T-cells in vitro. sGPC3 bound to CAR T-cells but failed to induce the activation of CAR T-cells, leading to an inhibitory effect on CAR T-cells in HCC.

Alpha-fetoprotein (AFP), a secreted glycoprotein, is abundantly expressed in the fetus and declines after birth; therefore, its elevation indicates an underlying pathology including hepatic disorders and malignancies, such as HCC [88]. The limitation with the CAR T-cells is that they can merely recognize tumor surface but not intracellular antigens. In addition, considering the fact that all intracellular antigens are presented by MHC class I molecules, the researcher generated some unique CAR T-cells that particularly bound to AFP 158–166 peptide-MHC complex lysing HLA-A*02:01+AFP+ tumor cells. This CAR T-cell therapy was used in AFP-expressing HCC patients [89].

Another study generated dual-targeted CAR T-cells against c-Met and PD-L1 [90]. Generally, c-Met is involved in hepatocyte proliferation, survival, and regeneration, though overexpression can endorse the development and progression of HCC [91]. PD-L1 negatively regulates the immune response by blocking the T cell receptor and CD28 signaling and thus is sufficient in immune evasion by suppressing anti-tumor immunity; therefore, dual-targeted CAR T-cells were effective toward c-Met+ PD-L1+ HCC cells and consisted of enhanced toxicity [92].

#### 3.1.3. Tumor-Infiltrating Lymphocytes

Tumor-infiltrating lymphocytes (TILs) in HCC tissue primarily contain diverse lymphocyte subgroups including T cells, B cells, and NK cells that possess significant roles in the development, treatment, and prognosis of HCC [93]. These cells can recognize several tumor antigens, as they are isolated from surgical tumor specimens and can be predictive of overall survival as well as disease-free survival after resection of both primary and metastatic liver tumors [94]. The tumor inhibitory effect of TILs is more robust than other therapies targeting single antigens or mutations. The viability of adjuvant TIL therapy has been demonstrated in phase III clinical trials in patients with HCC [95].

#### 3.1.4. Dendritic Cell Therapy

Dendritic cells (DCs) are capable of regulating cell-mediated immune responses by inducing activation and proliferation of antigen-specific cytotoxic T cells. However, their functions are severely impaired in HCC as these cells primarily constitute an immature phenotype with lower HLA-DR expression, decreased amount of IL-12, higher nitric oxide, as well as TNF-α production, resulting in poor capacity to induce allogeneic T cells [96]. DC-based immunotherapies offer a practical basis for tumor vaccines, tested in various malignancies including HCC. Ex vivo pulsed DCs with HCC cell line (HepG2) lysate presented anti-tumor ability in advanced HCC patients [97]. Since no single antigen is universally present in HCC, DCs pulsed with multiple tumor-associated antigens (TAA) including AFP, GPC-3, and MAGE-1 were reported to induce HCC-specific immune response [98,99]; although their clinical efficacy needs to be validated in a larger cohort of patients. While most of these practices stimulate mature DCs with TAA-derived proteins, peptides, or tumor lysates before injecting them, DCs can be administered intra-tumorally without extra stimulation by antigens [100]. However, techniques using antigen-derived peptide and proteins impose limitations in inducing broad immune response; therefore, the fusion of tumor lysates with DCs increased strongly to develop tumor vaccines due to its potential to induce the broad spectrum of anti-tumor immunity against unknown antigens and their T cell epitopes. Additional approaches comprising the re-administration of TAA-specific T cell upon stimulation with ex vivo induced DCs, as well as re-administration of DCs and CIK cell into the body after co-stimulation, are also being used and appeared safe and efficacious in enhancing anti-tumor immunity, improving survival rate, and survival time of HCC patients [101]. Moreover, DC-CIK seems to be effective in the treatment of HCC patients by controlling disease progression and complete remission in these patients; however, only a few faced partial remissions. DC-CIK acts on the growth cycle by inducing the expression of pro-apoptotic genes BAX and inhibiting the proliferation of cells by downregulating the proliferation of cell nuclear antigen (PCNA) gene in HepG2 cells, resulting in the inhibition of cell proliferation and HCC migration [102]. In fact, DC-CIK can induce the expression of other apoptotic molecules, such as caspase-3 leading to an enhanced apoptosis ratio through augmentation of caspase-3 and reduction in PCNA against liver cancer stem cells. Moreover, a streptococcus-derived tumor suppressor OK-432 can activate DCs, which is beneficial in enhancing anti-tumor activity [103]. HCC patients treated with transcatheter hepatic arterial embolization (TAE) alone and TAE plus OK-432 matured DC transfer, where inclusion of OK-432 induced DC maturation that led to higher expression of homing receptors, conserved phagocytic activity and markedly improved cytokine production, as well as tumoricidal activity [103]. Combination therapy prolonged the recurrence of free survival of patients in comparison to TAE monotherapy confirming that DC-based therapies benefit HCC patients. DC-CIKs combination with anti-PD-1 further boosted anti-tumoral effects and survival benefits by promoting the infiltration of immune cells into tumors. Therefore, blocking the PD-1/PD-L1 signaling pathway in DC-CIK cells before infusion is a better approach against HCC [104].

Alpha-fetoprotein serves as a promising target for the generation of DC-based vaccines. AFP-derived peptide-loaded DC vaccines enhance AFP-specific anti-tumor immune response by encouraging CD8 T cell response and NK cell activation, and lowering Tregs [105]. Several other antigen-loading approaches are associated with the effectiveness of DC-based vaccines. Recombinant adeno-associated virus (rAAV) can be safely used as a vector in gene therapy. Both rAAV-AFP-pulsed and cancer cell lysate-pulsed DC induce DC maturation, though rAAV-AFP-engineered DC vaccine emerged as superior to cancer cell lysate-pulsed vaccine due to its higher potential for T cell induction [106].

In addition, DC-derived exosomes, which are membrane driven vesicles, recently gained attention for vaccine therapy, providing a cell-free vaccine option for HCC treatment. Exosomes derived from AFP-expressing DCs (DEX_AFP_) elicited robust antigen-specific immune responses by enhancing IFN-γ and IL-2 production as well as lowering Tregs, IL-10, and TGF-β secretion in tumor region [107], resulting in the inhibition of tumor growth and protracted survival rates in mice with ectopic, orthotopic, and carcinogen-induced HCC, indicating DCs as a potential candidate for further therapeutic intervention [108]. Overall, we have summarized different cell-based therapies for HCC treatment in Table 1.

### 3.2. Use of Immune Checkpoint Inhibitors

Immune checkpoints are co-inhibitory molecules present on the lymphocytes, serving as an important regulator of T cell function, defining the balance between tolerance and autoimmunity. The regulatory function of different co-inhibitory molecules including PD-1, CTLA-4, TIGIT, TIM3, and LAG3 was initially revealed in the setting of autoimmune disease models where their blockade or deficiency caused induction or worsening of the disease [109]. Later, the presence of co-inhibitory receptors on lymphocytes was discovered to affect outcomes in chronic viral infections, tumors, and several cancers [110]. Liver tumors use this physiological mechanism to escape anti-tumor immune response by expressing the analogue ligands in tumor as well as stromal cells. Different immune cells express these inhibitory receptors. For instance, activated T cells, Tregs, NK cells, monocytes, and dendritic cells express PD-1, whereas a number of tumor cells, stromal cells, and myeloid cells [111] produce its ligand PD-L1/PD-L2. PD-1 is known to inhibit effector functions and leads to exhaustion and dysfunctional effector T cells. Similarly, CTLA-4 is displayed by the activated T cells and mostly Tregs, exhibiting different functions. It inhibits the activation of effector T cells while serving as an effector molecule for Tregs [112]. Atypical expression and function of immune checkpoint molecules has been observed in numerous cancers including HCC, where they impede anti-tumoral immune response, promoting tumor growth. The use of checkpoint inhibitors targeting PD-1 and its ligand PD-L1 and CTLA-4 emerged as tolerable and clinically beneficial in advanced HCC [82]. In fact, presently, the finest available first line of treatment for advanced HCC is a combination of PD-L1 blockade with atezolizumab and VEGF blockade with bevacizumab [113]. PD-1 and its ligand PD-L1 are the mainstay of systemic therapies in clinical practice. Immune checkpoint inhibitors are typically monoclonal antibodies, which inhibit the interaction between immune checkpoint proteins with their ligands averting the inactivation and functional exhaustion of T cells, restoring anti-tumor immunity. Anti-PD-1 monoclonal antibodies including nivolumab and pembrolizumab unambiguously appeared effective in different clinical trial settings when used alone or in combination with other checkpoint or tyrosine kinase inhibitors and VEGF/VEGFR inhibitors [114]. Nivolumab, a human IgG4 monoclonal antibody against PD-1 has proven overall survival benefits in patients with several types of cancers, such as metastatic melanoma, non-small-cell lung cancer, and advanced renal cell carcinoma [115]. Preliminary data from the checkmate-040 trial propose that nivolumab has clinical activity and is well tolerable in HCC patients including those with hepatitis B or hepatitis C virus infection [116]. Another randomized, multicenter phase III open-label study, CheckMate-459 (NCT02576509) compared the efficiency of nivolumab and sorafenib in patients with advanced HCC and reported median overall survival of 16.4 months with nivolumab as compared to sorafenib that showed overall median survival of 14.7 months [117], indicating that nivolumab treatment did not markedly improve overall survival in advance HCC patients compared to sorafenib; however, clinical activity and a favorable safety profile were observed. Moreover, the proportion of patients with grade 3–4 treatment-associated adverse events and any-grade treatment adverse events resulting in termination was lower with nivolumab than sorafenib. Therefore, nivolumab might be considered as an alternative therapeutic option in patients where tyrosine kinase inhibitors and anti-angiogenic drugs have significant risk and are contra-indicated. Other checkpoint inhibitors, such as PD-L1 and CTLA-4 can be potential targets to stimulate anti-tumor immune response [118]. Similarly, TIM3 and LAG3 are expressed on CD4 and CD8 TILs in HCC patients and negatively regulate effector T cell functions, providing evidence to investigate TIM3 and LAG3 inhibitors in HCC patients in combination with PD-1 and PD-L1 [119]. Table 2 summarizes the monoclonal antibody and combination therapy of checkpoint inhibitors for HCC treatment.

### 3.3. Use of Immune-Related Nanoparticles

Nanotechnology emerged as an advanced field in providing innovative prospects to overcome current challenges in HCC therapy, which is predominantly attributed to its unique approach for specific targeting, drug delivery, co-delivery of many drugs along with therapeutic properties of some nanomaterials themselves. Owing to its distinctive physical and chemical nature, nanoparticle-based platforms have been extensively used to regulate the immune system against cancer [120]. Nanoparticles can safeguard subtle antigens, proteins, and RNAs from being inactivated or degraded by enzymes present in the microenvironment and can be easily manipulated to accomplish effective immune cell regulation by attaching specific groups of antibodies, ligands, and peptides; therefore, they appear as a promising approach [121]. Macrophages are one of the most abundant cell types present in HCC tumor microenvironment where M2 type macrophages are favorably involved in immunosuppression, angiogenesis, tumor progression, and intrahepatic metastasis, indicating that preventing the infiltration of TAMs into tumor tissues and hindering their polarization into M2 type may be a potent therapeutic approach for HCC. Studies have identified that CXCR4, a G protein-coupled receptor, is involved in tumorigenesis by promoting M2 polarization owing to its higher expression in HCC due to sorafenib treatment [122]. CXCR4 antagonist, AMD3100, could inhibit cancer cell proliferation and M2 polarization by impeding the CXCR4/stromal cell-derived factor 1α (SDF-1α) axis. Therefore, a study that designed CXCR4-targeted lipid-coated poly lactic-co-glycolic acid (PLGA) nanoparticles of sorafenib modified with AMD3100 (ADOPSor-NPs) for targeted delivery of sorafenib to HCC, discovered that ADOPSor-NPs specifically deliver sorafenib to HCC, blocking CXCR4/SDF1α, and restrict M2 polarization and TAMs infiltration [122,123]; therefore, hindering tumor progression and improving overall survival in an in situ HCC mouse model. Moreover, TAMs infiltration is mediated by the chemokine ligands 2 and 5 that allow for their polarization into M2 phenotype, in order that the CCL2/CCL5 dual inhibitor (BisCCL2/5i) appears to be efficient in controlling their signaling pathways and endorsing macrophage polarization toward M1 phenotype, which was delivered using Dlin-MC3-DMA LNPs (MC3 LNP) to load mRNA encoding BisCCL2/5i [124]. The data have revealed that BisCCL2/5i-mRNA-LNPs treatment inhibited TAMs infiltration and induced the polarization of M2 macrophages in M1 type and improved the survival of experimental animals. Additionally, polydopamine nanoparticles have been synthesized to stabilize oxygen microcapsules (Oxy-Mic-Poly-Nano) to treat hypoxia in HCC patients as the presence of hypoxia can persuade the formation of inhibitory immune environment including the accumulation of TAMs and tumor progression [125]. Several lines of evidence indicate impaired anti-tumorigenic function in T cells. The designing of CD8- and glypican-3 antibody modified dual-target PLGA nanoparticles loaded with IL-12 could precisely bind to two target cell types including CD8+ T cells and HepG-2 cells via antibody–antigen interactions resulting in activation and proliferation of T cells by specific delivery of IL-12 into T cells [126]. Moreover, they empowered T cells to express higher levels of CD107a, improving its lytic activity to kill tumor cells via forming T cells and tumor cell clusters. Delivery of IL-12 mRNA using lipid nanoparticles allowed for IL-12 mRNA release into the cells where they were translated into protein using the ribosomes and contributed to HCC regression by activating the immune response. IL-12 lipid nanoparticles provoked the infiltration of activated CD44+CD3+CD4+ T cells into tumor and enhanced IFN-γ production suggesting that IL-12 LNP can serve as an effective immunotherapy against HCC [127]. Furthermore, there are nanoparticles that modulate the phenotype and functions of other immune cells, such as Tregs and DCs, which are being tested for the treatment of HCC. For instance, nanoliposomes encapsulated with H22 hepatoma lysate and conjugated with both mannose and CpG-ODN targeted mannose receptor on DCs to generate anti-tumor immunity, while CpG-ODN, as an adjuvant, elicited a strong immune response by enhancing the expression of costimulatory molecules and led to a smaller tumor volume and higher survival time in mice model compared with the control group. Additionally, MDSCs and Tregs potential culprits in the tumor microenvironment were declined, while IFN-γ and IgG levels were elevated [128], suggesting that these nanoparticles are efficient in generating robust humoral and cellular immune response.

## 4. Challenges and Future Directions

Despite extensive progress in HCC treatment, the present challenges cannot be ignored. The overall outcomes are still not satisfactory in terms of survival benefits, especially in the case of advanced HCC [129], which are not only not accredited to tumor but are associated with complications including variceal hemorrhage, hepatic encephalopathy, hepatorenal syndrome, and ascites contributing to substantial mortality [130]; therefore, requiring significant attention during the treatment strategy. The optimization of HCC treatment needs an individualized treatment plan considering different factors, such as (1) the condition of the patients and (2) their comorbid illness, (3) treatment options ranging from curative surgery for patients with early to advanced stage HCC along with metastatic condition. Moreover, the surveillance techniques are insufficient in ensuring a higher number of advanced HCC cases [131]; therefore, better surveillance is obligatory to detect the condition at an earlier stage, such as cirrhosis or chronic liver disease, in order to offer better prognosis and outcomes. Although the diagnosis based on non-invasive technique is confronted due to the necessity of molecular understanding of the disease that requires tissue specimen, in this aspect, a standardized routine for collecting the biopsies in clinical practice is evolving and is highly warranted. The evidence-based therapies are quite limited for HCC treatment, sorafenib is the only systemic drug that has been established to be clinically effective in treating advanced HCC for a longer period [132]. Furthermore, in the case of immunotherapies, it is hard to predict which patients would respond to therapy, probing the need of non-invasive biomarkers to anticipate a treatment response to immunotherapies.

In addition, the cost of HCC treatment imposes an economic burden that is indeed significantly higher than calculated since the cost is mostly calculated without considering patients’ comorbid conditions, which require treatment, increasing the cost of the treatment further [133]. Therefore, accounting for patients’ comorbidity would not only assist in calculating the approximate treatment cost precisely, but also benefit us in the identification of the comorbid condition, which creates an obstacle to HCC treatment. Moreover, the presence of underlying liver disease in most patients contributes to overall reduced survival. In these cases, there is a “point of no return” beyond which the liver will not revert, and a transplantation will be required.

## 5. Conclusions

Hepatic tumor immune microenvironment is complex and heterogeneous in nature, consisting of a wide variety of immune cell population involved in immune tolerance, growth, invasion, metastasis, and drug resistance in HCC. Given that host immune response is substantially critical, eliciting robust HCC-specific immune response represents a novel therapeutic approach to introduce more into clinical practice. However, due to limited treatment efficacy in some groups of patients, advancement in the understanding of immune response dynamics and complexity is further warranted to improve the future of immunotherapeutics in HCC.

## Figures and Tables

**Table 1:** Cell-based therapies for HCC treatment.

Cell Types	Patients Included (n)	Target	Phase	Trial Number
Anti-GPC3 CAR T-cells	38	Glypican-3 (GPC3 positive HCC)	I	NCT05003895
ET140202 genetically modified autologous T cells to carry a TCR mimic	2	Tumor-specific intracellular antigens	I and II	NCT03998033
Genetically modified autologous AFP^c332^ T cells	45	AFP-expressing tumors	I	NCT03132792
ECT204 autologous T cell therapy	12	-	I and II	NCT04864054
Autologous HBV-specific T cell receptor engineered T cells	36	-	I	NCT05339321
Expanded activated lymphocytes	430	-	II	NCT05213637
TCR-redirected T cells Therapy	10	-	I	NCT03899415
TACE combined with PD-1 knockout engineered T cells	10	-	I	NCT04417764
New antigen reactive cells (NRT) in combination with radiotherapy	40	-	IB/II	NCT03199807
Autologous gamma delta T cells	8	-	Early phase 1	NCT04518774
B7H3 CAR T-cells	5	B7H3+ cancer cells	I1	NCT05323201
Adoptive transfer of specific HCC antigens CD8+ T cells	18	-	I	NCT03175705
Adoptive transfer of iNKT cells	18	-	I	NCT03175679
Neoantigen-based dendritic cell vaccine	24	-	I	NCT03674073
DC-CIK	60	-	II	NCT02487017
DC vaccine	18	-	I	NCT01974661
DCs coactivated by HBV-specific antigen peptides and HepG2 cell lysate	70	-	I and II	NCT03086564
Cyclophosphamide and multiple signals-loaded dendritic cell vaccine	600	-	II	NCT04317248

**Table 2:** Mono and combination therapy of checkpoint inhibitors for HCC treatment.

Drug	Patients Included (n)	Target	Phase	Trial Number
**Monotherapy**				
Anti-PD-1 antibody Pembrolizumab	30	PD-1	II	NCT03419481
Nivolumab	743	PD-1	III	NCT02576509
Nivolumab	659	PD-1	I	NCT01658878
Camrelizumab	20	PD-1	I	NCT04564313
**Combination therapy with two immune checkpoint inhibitors**				
TSR022 and TSR042	42	TIM3 and PD-1	II	NCT03680508
Nivolumab plus Ipilimumab	40	PD-1 and CTLA-4		NCT03510871
Nivolumab with and without Relatlimab	20	PD-1 and LAG-3	I	NCT04658147
Durvalumab plus Tremelimumab	30	PDL-1 and CTLA-4	II	NCT03638141
**Combination therapy of immune checkpoint inhibitors with tyrosine kinase inhibitors**				
Telipril plus Apatinib in combination with SBRT	20	PD-1 and tyrosine kinase	II	NCT04165174
Camrelizumab and Rivoceranib	482	PD-1 and tyrosine kinase	III	NCT04639180
Toripalimab (JS001) plus Lenvatinib	519	PD-1 and tyrosine kinase	III	NCT04523493
Sintilimab plus Anlotinib	20	Tyrosine kinase and PD-1	II	NCT04052152
Camrelizumab plus Rivoceranib	674	PD-1 and tyrosine kinase	III	NCT04639180
Pembrolizumab plus Regorafenib	95	PD-1 and multiple kinases	II	NCT04696055
**Combination of immune checkpoint inhibitors with VEGF/VEGFR inhibitors**				
Atezolizumab plus Bevacizumab	434	PD-L1 and VEGF	III	NCT4803994
Atezolizumab plus Bevacizumab	45	PD-L1 and VEGF	II	NCT044954339
TQB2450 and Anlotinib	70	PDL-1 and VEGFR	II	NCT05311319
HX008 in combination with Bevacizumab or Lenvatinib	72	PD-1, VEGFR, and VEGF	II	NCT04741165
AK104 with and without Lenvatinib	75	Bispecific for PD-1 and CTLA-4, VEGFR	II	NCT04728321
KN046 combined with Lenvatinib	55	Bispecific for PD-L1 and CTLA-4, VEGFR	II	NCT04542837
**Combination of immune checkpoint inhibitors with chemokine inhibitor**				
Nivolumab combined with BMS-986253	74	PD-1 and IL-8	II	NCT04050462
**Combination of immune checkpoint inhibitors with non-invasive/minimally invasive procedure**				
Sintilimab and TACE	41	PD-1	II	NCT04842565
Pembrolizumab plus RFA	30	PD-1	II	NCT03753659
Anti-PD-1 antibody and RT	39	PD-1	II	NCT04193696

## Data Availability

Not applicable.
